# Six Years of Sick Leave Spells in a Group of University Civil Workers. Can Modern Work Bring Them a New Health Problem?

**DOI:** 10.3390/ijerph16010017

**Published:** 2018-12-21

**Authors:** Adriano Dias, João Marcos Bernardes, Miriam Malacize Fantazia, Carlos Ruiz-Frutos, Juan Gómez-Salgado

**Affiliations:** 1 Public Health Grade Program, Botucatu Medical School, Paulista State University, Botucatu, Sao Paulo 18618687, Brazil; dias.adriano@unesp.br (A.D.); jmbernardes@yahoo.com (J.M.B.); adrianodiass@hotmail.com (M.M.F.); 2 Department of Sociology, Social Work and Public Health, Universidad de Huelva, 21071 Huelva, Spain; 3 Department of Nursing, Universidad de Huelva, 21071 Huelva, Spain; jgsalgad@gmail.com; 4 Safety and Health Posgrade Program, Universidad Espíritu Santo, Samborondón, Guayaquil 091650, Ecuador

**Keywords:** sickness absence, public workers, university, psychosocial work environment, occupational health, health workers

## Abstract

The objective of this study is to analyse sick leave episodes of a university’s collective of statutory workers in the State of São Paulo, between January 2010 and December 2015. For this, a descriptive study analysed 5776 registered spells of sick leave of four university units: agricultural sciences; human health, health and animal reproduction, and biological sciences; an administrative unit; and a university hospital. The medical expert assessment was carried out by general practitioners and psychiatrists who managed sick leave and return to work cases. Around 52% had up to three sick leave episodes, and 10% of the workers had 20 or more episodes. Each spell of sickness absence lasted a median of 30 days (IQR 8–60 days). Among all of sick leaves, 35% had as a primary cause mental or behavioural diseases, of which 30% were depressive disorders, followed by around 18% related to the musculoskeletal system and the connective tissues. In the medical reports, 80% of the workers reported pain and 30% reported psychological symptoms. The collective, seen as privileged by many for their job stability, has a high percentage of sick leave due to mental illness, with extended periods which affect the levels of disability and reduce possibilities of return.

## 1. Introduction

Sickness absence is a complex phenomenon that is strongly influenced by factors other than health [1,2,3]. It has been suggested that many workers will attend work with conditions that others will be absent with, and that where there are high rates of absence there are also high rates of “presenteeism”. Many EU governments have introduced programmes aimed at encouraging long term absentees getting back into the workforce [1].

In Brazil, civil servants may be statutory or public employees. The former are holders of public office and are subject to statutory regimes established by the federal, state and local governments. On the other hand, the latter are subject to the rules of the Consolidation of Labour Laws (CLT), the decree that governs labour relations in Brazil [4].

In Brazilian public universities, the same rule coexists. However, the workers are divided into two other categories, technical-administrative and professors. Also, there may additional differences between the weekly work time, which may be full or part-time, and the type of dedication, exclusive or not. Thus, Brazilian universities have a multiplicity of worker types, not to mention outsourced workers, who deserve a separate analysis.

As many as 12% of Brazilian workers are civil servants, a similar proportion to some Latin American countries, but far from the percentages found in developed countries, where the average is 21%, according to the Organization for Economic Co-operation and Development [5]. It is even further from some Scandinavian countries where more than a third of the economically active population is employed in the public service.

Even though working as a civil servant requires passing a civil service examination, it is highly desirable. This is due to the security and employment stability provided by the organisations, opportunities for professional formation and qualification, possibilities of professional advancement and salary increases through career plans and promotions, coverage of special pension regimes with higher pensions for lower contribution rates [6], while workers in the private sector tend to lead unemployment statistics. As a consequence of the higher level of education needed to be admitted to public service and its development during working years, more than half of civil servants have a higher education degree. On the other hand, in the private sector this percentage is 15% [7]. For example, more than 60% of the employees of a public university have a higher education degree, even though they occupy high school education level positions [8].

Although these benefits still exist or at least part of them, from the 1990s onwards and parallel to the transformations that occurred in business, the Government also reformed itself. It did so through a politico-ideological movement with economic roots, adopting management practices, until then typical of the private sector, and focusing on the search for quality and efficiency [9,10,11,12,13].

The changes in technical devices, management rules and the reorganisation of work environments resulted, for the workers, in an increase in qualification, workload and flexibility, in addition to the reduction of autonomy and remuneration. At the same time, this process accentuated competitive behaviours and disarticulated the capacity for social support, producing feelings of isolation and insecurity in employment [14,15]. In addition, the public service extinguished positions considered non-essential, allowing the hiring of outsourced workers (for a specific period of time, under different contract regimes and with unequal/lower salaries), of whom compliance with goals and an increase in pace and productivity was demanded [15].

A non-ideal work environment was generated as a result of all these changes, increasing psychosocial risk markers for workers’ physical and mental health problems: excessive workloads, conflicting work demands, lack of clarity of function, lack of involvement in decision making and of influence on the way work is done, insecurity, ineffective communication and lack of social support from supervisors or colleagues [16,17]. These risk factors affected in quantity and intensity, what contrasts with the improvements in general health conditions of the world population in the same period [18] and, mainly, with the healthy worker effect [19].

The main indicator of workers’ health problems is absenteeism [20], here defined as absence to work due to a disease and justified by a medical statement [21]. Studies on absenteeism in civil servants [22,23,24,25,26,27] indicate that the predominant disease is related to work activities and the professionals involved; and, also, that they are more frequent in female workers and in the age group between 30 and 45 years. Between 2003 and 2006, according to data from all civil servants employed by all departments and agencies related to the Government of São Paulo State (Southeast Brazil), nearly 200,000 sick leaves were granted each year, with mental disorders being responsible for almost 30% of these leaves [22].

In Brazil, official data [28] shows that mental disorders represent 10% of all pension benefits, being surpassed only by musculoskeletal disorders (20%) and external causes (25%). This is certainly resulting from the negative effects of these new work environments on workers’ health.

In addition to the impact of absenteeism on productivity and interpersonal relationships at work, workers’ illness often results in long-term sick leave. This implies difficulties in returning to work, mainly when it involves changes in job, function and the status derived from the limitations caused by the disease.

Thus, the objective of this study is to present an epidemiological profile of a university group of civil servants’ sick leaves over a period of six years, as well as defining work characteristics and possible causes of sick leave.

## 2. Materials and Methods 

### 2.1. Study Design and Setting

This is a descriptive study of sick leaves. Its population was composed of civil servants from a university located in inland São Paulo State, Brazil, that took sick leaves between the months of January 2010 and December 2015.

During the period that the information was collected by the University’s Technical Section of Worker’s Health, the university campus was composed by four academic units (agricultural sciences, human health, biological sciences and animal health and reproduction), an administration unit, and a university hospital. 

### 2.2. Participants

The sum of the populations in these units, in the middle of the period (2012), was 4090 workers, among which about 60% were statutory civil servants, while the others were public employees subject to the CLT or outsourced public workers. The approximate proportional distribution of workers per unit, in the middle of the period, was: administration (5%), agricultural sciences (13%), human health and hospital (56%), animal health (11%), biological sciences (15%).

During the six years of the study, 965 workers were examined by physicians, resulting in 5776 episodes of sick leaves, which comprised the study sample. It should be noted that the estimates of the populations made available from each year were fluctuating, which made it difficult to establish precise annual denominators and imposed the need to analyse the data globally.

The institution has its own occupational medical service, who examines all statutory workers who request a sick leave with duration of two days or more. This service is composed by general practitioners, who evaluate sick leave applications due to non-psychiatric morbidity, and psychiatrists, who evaluate sick leave applications due to mental disorders. The service not only manages all sick leaves applications, since it is the only one that can grant or deny them, but also all cases of readaptation to work.

### 2.3. Variables and Data Sources

For the exploratory phase of the study, data was extracted from two institutional databases: (a) Integrated Occupational Management Software (IOM) (UNESP, São Paulo, Brazil), which identifies the worker and stores physician’s evaluation data; (b) Healthcare Medical System (HMS) (UNESP, São Paulo, Brazil), which records information about cases of work readaptation.

It is important to highlight that the two databases were not integrated or built under the same computational architecture (neither for research purposes), nor did it allow searches or information filtering (in the case of the HMS), which made it necessary to evaluate one by one all cases of work readaptation already made by that service to make them coincide with the valuations of the period.

Then, after retrieving and organising the information, a database was built. It contained variables of the following types: sociodemographic (place of birth, sex, current age and age when hired by the university, marital status and coexistence with partner), job characteristics (position, work unit, rehabilitation history, working time in the institution and total work time), sick leave characteristics (duration of each episode, number of medical evaluations in the period, total duration of all episodes, reason of the leave classified by the International Classification of Diseases (ICD-10) and behaviour of repeated sick leaves) and work readaptation (if it happened, time passed until it happened and if it resulted in any limitations). The reason for each sick leave was defined as the first or main diagnosis pointed by the physician, since it could have multiple reasons. Thus, it was necessary to create data mining tools to search for the reason of each leave and for variables related to the physicians’ evaluations (type of injury, affected part and presence of psychological symptoms) in the IOM, a textual qualitative record base.

### 2.4. Statistical Analysis

The variables were analysed using simple and cumulative percentage distributions and measures of central tendency and dispersion (for discrete and continuous variables, respectively), using IBM SPSS software, v.20.0 (IBM, Armonk, NY, USA).

### 2.5. Ethical and Legal Aspects

The research has been carried out with the consent and support of the Human Resources of the units involved and was approved by the Ethics Committee of the Botucatu Medical School (protocol 1.874.625, of 12/19/2016).

## 3. Results

A total of 5776 spells of sick leave, of two or more days, from 965 workers, were studied. Table 1 shows that workers who took a sick leave had been hired by the university at a young age (28.6 ± 6.7 years), but they had been in the institution for a long time (21.1 ± 7.6 years) before taking the first sick leave during the period studied. 

The majority were women (62.7%), lived with a partner (55.8%) and born in the State of São Paulo (98.7%). The largest proportion of workers belonged to the Human Health university unit (77.5%) and occupied mid-level healthcare positions (38.8%).

Table 2 shows that the population had, on average, about 50 years of age when they took the sick leave and that the average total duration of the sick leave per worker was 564 days, varying between 15 to more than 6 thousand days, distributed in single (23.5%) and multiple (76.5%) leaves. It should be highlighted that 23.5% and 17% of the workers had had one and two spells of sick leave, respectively, and almost 80% of the workers had up to 10 episodes during the study period. 10% had 20 or more, reaching a maximum of 59. Each spell of sick leave (n = 5776) had a median length of absence of 30 days (IQR 8–60 days), with minimums and maximums of 2 and 1439 days, respectively.

More than one third (35%) of all sick leaves had, as primary (or main) cause, diseases classified as mental or behavioural disorders, according to the ICD-10 [29]. These were followed at a distance by diseases of the musculoskeletal system and of the connective tissue (17.8%). According to the medical evaluations, 20% of all cases affected the trunk; there was pain in up to 80% of the episodes; and there was psychological symptomatology in 30% of all cases.

Regarding the main cause according to the ICD-10, considering up to the 20^th^ episode of sick leave (91.5% of cases), the cause of the first episode was modified in subsequent events in 45% of the cases, and it may have or not returned to the cause of the first episode. The main cause was subsequently modified to a diagnosis included in the chapter ‘Mental and behavioural disorders’ from the ICD-10 in 20% of the sick leaves that did not have a mental disorder as the main cause in the first episode. And, in about 23% of the cases, mental disorders were the main cause from the beginning until the end of the follow-up.

Table 2 shows that 26% (239) of the 965 workers who took a sick leave had to be readapted to work, with an average waiting period of 344 days. Of these, 77.7% (195) were readapted with some degree of activities’ limitation when returning to work.

Table 3 presents the 51 most frequent causes of sick leave among the 150 diseases that were registered as the main cause in the period studied and that represent 90.1% of the total causes. Among the mental and behavioural disorders (Chapter V of the ICD-10), we see depressive disorders and the use of psychoactive substances as those that were most frequently recorded. Meantime, among the osteomuscular diseases (Chapter XIII of the ICD-10), dorsalgias, arthropathies and shoulder diseases were the most frequent. The other 99 diseases corresponded to 9.9% of the events among the 5776 registered spells of sick leave.

## 4. Discussion

First of all, it should be highlighted that the databases accessed to obtain the information used in this research contained data only on the civil servants who took at least one spell of sick leave during the study’s period. Therefore, our results and their interpretation are valid only to this particular group of individuals and cannot be generalised to university civil servants in general.

The present study allows us to describe sick leave causes in a group of great importance because of its number and that is not usually studied, such as public university workers. This is a group of workers seen by many as privileged in terms of psychosocial risk protection due to their special work regime (for the most part of them), their job stability [30] and/or their educational levels above the average [7]. We identified a high percentage of sick leaves related to mental disorders (35%), of which 30% are depressive disorders. Having data on the causes, frequency and duration of sick leaves makes it possible to propose preventive measures related to organisational factors and aimed at improving the management of health services at these public institutions.

It must be borne in mind that the results presented in this study refer only to a subgroup of civil servants, statutory workers. However, the fact that the results show that workers with a medium level of education are the ones who take more sick leaves seems to corroborate the statement of health protection for that kind of worker. At the same time, it should be considered that these workers occupy lower positions in the employment hierarchy and perform less flexible activities, while those with a higher level of education tend to occupy management positions. These positions are frequently more flexible, and may be able to make use of informal agreements. Thus, their sick leaves may be under-registered in the system.

When a civil servant (regulated by statute or specific regulation, contributor of a provisional regime that belongs to an administrative unit, whether federal, state or municipal) requests a sick leave, he/she undergoes a medical expert examination that aims at the social and financial equilibrium of the institutions [31]. This study was conducted at an institution where the medical evaluations of sick leave are made by physicians who depend on the same employer of the workers they examine. This could mean that their decisions may not correspond to the reality of the diseased workers, for example, anticipating times of return to work. Although the medical evaluations carried out by these professionals may induce doubts, it is evident that the centralisation of these evaluations at the institution’s own occupational medical service leads not only to reductions of travels to undergo examinations, but also facilitate the standardisation of criteria [32].

The expected work time until a worker takes a sick leave should be in accordance with the life cycle, in other words, older workers should be more likely to take sick leaves. This statement is corroborated in the present study, in which we see that 76% of workers were over 46 years of age and the average time they had spent in the institution until the first sick leave was over 21 years. However, other international studies on sick leaves due to non-work directly related causes show that this proportion is less than 30% [33].

Another finding of this research is that almost two thirds of the workers who took a sick leave were women, while in other scenarios women were less than 50% [33]. Consideration should be given to sex discrimination, which could influence the distribution of medium and higher education positions and its relation to the frequency of sick leaves. Although when analysing only sick leave cases without having the denominator of exposed workers, we cannot affirm that the proportion in one of the two sexes is greater or not. Likewise, it is well known from previous studies that women must attend to family emergencies to a greater extent than men, and this generates a higher percentage of sick leaves because there is no other mechanism that allows an individual to take care of children or other family members when such events arise [34].

The university unit with the highest proportion of sick leaves was the Human Health one (77.5%), in equal percentage to the proportion of workers of the Faculty of Medicine, and the university hospital accounted for 73% of those who composed this study’s sample. It should be highlighted that almost half of the sick leaves were taken by health care workers, a fact that corroborates that, due to risks ranging from biological to organisational type [35,36,37], they are a population of workers quite vulnerable to temporary incapacities for work. 

Making a reflection on the two previous considerations, the higher prevalence of workers from the Human Health unit can also determine the higher incidence of sick leaves among women, since the majority of the workers in the health sector are females, as stated by international and Brazilian literature [38,39,40].

The average duration of sick leaves for each worker probably varies in function of age, the type of work carried out and seriousness of the disease, associations that were not analysed since this is a descriptive study. However, it is necessary to highlight the multiplicity of events that the same worker can have, since 77% of the workers had more than one episode of sick leave in the study period and, of these, 45% had repeated episodes, maintaining the main cause from the first to the last episode. That is to say, it is very possible to infer tendencies of chronification of the diseases that originated the sick leaves, although they can also be explained, in part, by the greater age of the studied workers. However, to corroborate these hypotheses, no studies were found that had investigated predictors of the multiplicity of sick leaves.

Analysing chronic diseases, if we assume that longer sick leaves are a good example, we observe that these occurred to a greater extent among mental and behavioural diseases. This is consistent with the natural history of these diseases given that this type of pathologies produces acute symptoms to a lesser extent, except in some cases such as depressive disorders. However, if we analyse the records of the medical evaluations in less than a third of the registered sick leaves, psychological symptoms were identified as a complaint.

On the other hand, the same medical evaluation reports (when based on the initial complaints) that initially did not corroborate the diagnoses of the causes of temporary incapacity for work, now revealed that pain was present in 80% of all events. Thus, it is a widely found symptom in the majority of the acute causes that originated temporary incapacities for work, although they can also become chronic.

The finding that more than a third of the sick leaves had as their main cause mental and behavioural diseases is an important warning, especially because it does not agree with other populational studies [28]. In these, they are the fourth cause (and very close to the fifth), with a percentage close to 9% of the total. This was not either in accordance with other European studies where this group of diseases is the fifth cause and accounts for less than 8% of the sick leave total, behind musculoskeletal, respiratory, infectious diseases or those grouped in chapters XIX and XX of the ICD-10, generically described as external causes [32,33].

Depressive disorders were the most prevalent among all registered illnesses and one fifth of the workers had a main diagnosis related to mental health throughout their sick leave history. These results are congruent with previous studies [41] and can be explained by several reasons, which may also interact. We see how the mental health of civil servants is worse than that of the workers of the private sector. This leads us to believe that the recent changes in management mechanisms and work organisation that the civil sector has been through have led to work organisation models typical of the private initiative, based on productivity, which may impact more significantly on civil servants than in other sectors.

Another possible explanation is that the presence of psychiatrists among the physicians that work in the institution’s own occupational medical service could lead to a better diagnosis or overdiagnosis of mental and behavioural diseases. It has been described, in previous publications, that the presence of such specialists can increase the diagnoses of mental illnesses and that such inadequate diagnoses and treatments can increase the risk for the said persons to develop physical disorders such as diabetes, cardiac disorders, weight gain, and other potentially serious health conditions [42].

In any case, due to overdiagnosis or not, addictions as a consequence of mental and behavioural disorders imply a large number of sick leaves to require medical treatment, in addition to great personal, institutional, economic and social losses, such as those derived from absenteeism, reduction of work ability and loss of productivity [15,22,26]. Substance addiction is also associated with jobs with high psychological demands and low work control [43,44], and the high prevalence obtained in this study can indicate that the work environment in the university presents such negative characteristics to workers’ mental health.

There is consensus on the high cost of mental illness attributed to the loss of productivity and measured as absenteeism or lost work days. Thus, it is advisable to open lines of research that allow us to better understand the incidence, extent and recurrence of this type of diseases in this workers’ population [45].

A large number of workers needed a readaptation of their previous work activity and, for many of them, with limitations. This aspect is important because we know from bibliography that the longer the duration of the sick leave the more it is related to the degree of disability and the lower the probability of returning to their original job [46].

### Strength and Limitations

There are some limitations in this study. First, sick-leaves are multi-factorial and influenced not only by the health status of the individuals, but also by their work environment, social and psychological factors [47], attitudes and commitment to work as well as social insurance system. Thus, since the medical reports mainly contained health related information, we cannot make inferences about other possible sick leave causes related, for example, to psychosocial and workplace factors.

Second, it is a common problem for many registry-based studies of sick leaves that they only have access to one diagnosis, while we know that workers often struggle with several complaints and illnesses. Unfortunately, this was also the case of the databases that we had access to. They did not contain all the information we consider relevant, such as all the diagnoses related to each sick leave spell. In the system, only the main diagnosis of each sick leave spell is recorded. To identify all the workers with multiple complaints and their diagnoses, access to the medical records of each worker would be needed. However, we did not have access to these documents.

Third, we did not have access to the total number of workers at the units, forbidding the possibility to establish a denominator for the amount of sick leaves. This is a clear limitation, as it precludes us to know e.g. the overall prevalence and/or incidence of sick leaves in the units or to what extent the distributions of the various sociodemographic and work characteristics mirror that of the working population as a whole. The total number of workers at the units was not known, since it was fluctuating (due to hiring, layoffs, transfers and retirements), both during the entire study period and yearly. This made it difficult, if not almost impossible, to establish a precise estimation of the workers’ population (needed denominator for prevalence and incidence calculation), and the decision to run an overall analysis of the entire six-year period made it even harder. A possible solution would be to use the number of workers recorded in the middle of 2012 (2364). However, this would bring even more inaccuracies and lead to the stratification of the analysis year by year, what would end up determining an annual analysis. Fourth, our results may be conditioned by the fact that the medical evaluations of sick leaves are made by physicians that work in the same university institution, being able to speed the return to work. However, a strength related to this centralisation of the evaluations, by the institution’s own occupational medical service, is the standardisation of criteria.

The main difficulty to perform this study was that both databases were not integrated or built under the same computational architecture. This fact forced us to build the database used in the study manually, which was time-consuming and required extensive effort to collect information. These databases also presented most of the five methodological problems regarding the analysis of sickness absence described by Hensing et al. [48]. Solving these problems would facilitate the use of this useful source of information, not only for research purposes but also for the follow-up of sick leaves by the occupational medical service in the future.

## 5. Conclusions

Around 90% of all spells of sick leave were due to mental illness and musculoskeletal disorders. We would highlight that, when compared with other groups of sick workers, they have a higher number of sick leave spells due to mental illnesses, mostly depressive disorders. This fact has a negative effect on the workers involved and higher costs for the university. This is because they are a type of sick leave associated with higher levels of disability, fewer possibilities of return to work and that needs a process of readaptation to the previous job position. From a practical perspective, these results are of great relevance for those involved with workers’ health management: they allow an overview of the diseases that are related to sick leaves in university workers, their burden, and also the widening of Occupational Health and Safety (OSH) managers’ understanding about workers’ sociodemographic data relation with sick leaves and return to work. Furthermore, the results might guide the construction of a workplace’s preventive and protective measures and of health promotion interventions to reduce the burden on workers’ health.

Using both record databases (Integrated Occupational Management Software and Health Medical System) to acquire the data used in this paper has allowed us to identify some points that must be improved in order to achieve the full potential of these tools, such as the absence of important data, the use of different computational architecture for both databases and the need of linkage between them. But this is not all, they have allowed us to demonstrate the usefulness of these databases for better sick leaves management by OSH managers and workers.

The analysis of data on sickness absences stored in computerised databases can lead to the detection of failures in the management system, the identification of groups, pathologies and organisational measures where action is needed. An example is the greater vulnerability found in the “Human health” unit that can be explained by being composed mostly by females and health personnel, two of the variables that in previous studies have been associated with a greater number of sick leave spells. 

Finally, the effort to extract the data from both databases and reconstruct it under a unified database will contribute for OSH researchers and workers in concomitant and/or subsequent studies. With these, we intend to investigate some hypotheses that were generated during this study regarding predictors of multiple spells of sick leaves. For example, the ageing of the workforce due to not replacing retired workers and the effects of the economic crisis.

To conclude, the studied sick leaves taken by the university workers differ from those presented in previous publications, since more than a third of the sick leaves had as their main cause mental and behavioural diseases, also because women comprised almost two-thirds of the workers who took a sick leave and due to the fact that 76% of the workers who took a sick leave were over 46 years old, as previously stated in the discussion. Furthermore, the long duration of the sick leaves found in this study is remarkable and it may be related to the fact that multiple repetition of events occurred in several cases.

We would also like to highlight the important contingent of workers that were readapted to their previous work activity and the high percentage of those who were readapted to work with limitations. This is important since it is well known that the longer the sick leave is, the higher the disability levels and the lower the chances of return to work.

## Figures and Tables

**Table 1:** Sociodemographic and working characteristics of the study population (n = 965).

	Mean	SD	Min.	Max.	p25	Median	p75
Age started working at university	28.65	6.74	18.35	59.22	23.07	27.84	33.10
Time working at university	21.12	7.65	0.4	50.39	17.02	21.73	25.99
					n	%	C % *
Sex	Female				605	62.7	62.7
	Male				360	37.3	100.0
Marital status	Married				538	55.8	55.8
	Separated				232	24.0	79.8
	Divorced				132	13.7	93.5
	Single				57	5.9	99.4
	Widowed				6	0.6	100.0
Has a partner	Yes				538	55.8	55.8
	No				427	44.2	100.0
Origin	São Paulo				952	98.7	98.7
	Another state				13	1.3	100.0
Unit	Administration				41	4.2	4.2
	Agricultural sciences				61	6.3	10.6
	Human health				748	77.5	88.1
	Animal health				56	5.8	93.9
	Biological sciences				59	6.1	100.0
Position	Mid-level healthcare				374	38.8	38.8
	Administration				143	14.8	53.6
	Operational				119	12.3	65.9
	Academic support				72	7.5	73.4
	High-level healthcare				61	6.3	79.7
	Teaching				54	5.6	85.3
	Rural work				45	4.7	89.9
	Others high-level				22	2.3	92.2
	Supervision and reception				22	2.3	94.5
	Others mid-level				16	1.7	96.2
	Supervisor				15	1.6	97.7
	Radiotherapy				13	1.3	99.1
	Transport				9	0.9	100.0

* cumulative percentage.

**Table 2:** Characteristics of sick leaves.

	Mean	SD	Min.	Max.	p25	Median	p75
Age at the start of the process (n = 965) #	49.77	7.67	25.58	81.61	45.28	50.01	54.68
Total length of absence in days (n = 965)	564.47	1053.95	15.00	6077.00	79.00	147.00	351.00
Time until readaptation in days (n = 239)	343.24	684.99	2.00	5925.00	15.00	60.00	290.50
Each sick leave spell length of absence (n = 5776)	50.19	129.27	2.00	1439.00	8.00	30.00	60.00
					n	%	% cum.
Readaptation (n = 965)	No				714	74.0	74.0
	Yes				251	26.0	100.0
Readaptation with limitations (n = 251)	No				56	22.3	22.3
	Yes				195	77.7	100.0
Number of medical evaluations in the period (n = 965)	1				227	23.5	23.5
	2				162	16.8	40.3
	3				111	11.5	51.8
	4				73	7.6	59.4
	5				58	6.0	65.4
	6–10				140	14.5	79.9
	11–20				112	11.6	91.5
	21–59				82	8.5	100.0
ICD-10 chapter (n = 5776) *	I				130	2.3	2.3
	II				219	3.8	6.0
	III				13	0.2	6.3
	IV				83	1.4	7.7
	V				2022	35.0	42.7
	VI				131	2.3	45.0
	VII				215	3.7	48.7
	VIII				48	0.8	49.5
	IX				245	4.2	53.8
	X				182	3.2	56.9
	XI				189	3.3	60.2
	XII				96	1.7	61.9
	XIII				1031	17.8	79.7
	XIV				168	2.9	82.6
	XV				22	0.4	83.0
	XVI				1	0.0	83.0
	XVII				18	0.3	83.3
	XVIII				89	1.5	84.9
	XIX				362	6.3	91.1
	XX				25	0.4	91.6
	XXI				487	8.4	100.0
Injured body part (n = 1018) **	Trunk				228	22.4	22.4
	Hands				162	15.9	38.3
	Eyes				151	14.8	53.1
	Feet				130	12.8	65.9
	Arms				124	12.2	78.1
	Legs				99	9.7	87.8
	Head				87	8.5	96.4
	Fingers				37	3.6	100.0
Type of injury (n = 1385) **	Pain				1112	80.3	80.3
	Fracture				141	10.2	90.5
	Contusion				55	4.0	94.4
	Sprain				35	2.5	97.0
	Burn				17	1.2	98.2
	Sharp-cutting				15	1.1	99.3
	Dislocation				8	0.6	99.9
	Lethargy				2	0.1	100.0
Psychological symptoms (n = 5776) **	No				4044	70.0	70.0
	Yes				1732	30.0	100.0
Symptoms start with other chapter and change to chapter V of the ICD-10 until 20^th^ expert record (n = 965)	No				768	79.6	79.6
	Yes				197	20.4	100.0
Symptoms start with chapter V of the ICD-10 and is kept until 20^th^ expert record (n = 965)	No				740	76.7	76.7
	Yes				225	23.3	100.0
ICD chapter modified (n = 965)	No				332	45.0	45.0
	Yes				406	55.0	100.0

# Refers to the worker’s age at the first sick leave spell during the study period. * I—Certain infectious and parasitic diseases; II—Tumours (Neoplasms); III—Diseases of the blood and blood-forming organs and certain disorders involving the immune mechanism; IV—Endocrine, nutritional and metabolic diseases; V—Mental and behavioural disorders; VI-Diseases of the nervous system; VII—Diseases of the eye, adnexa. VIII—Diseases of the ear and mastoid process; IX—Diseases of the circulatory system; X—Diseases of the respiratory system; XI—Diseases of the digestive system; XII—Diseases of the skin and subcutaneous tissue; XIII—Diseases of the musculoskeletal system and connective tissue. XIV—Diseases of the genitourinary system; XV—Pregnancy, childbirth and the puerperium; XVI —Certain conditions originating in the perinatal period; XVII—Congenital malformations, deformations and chromosomal abnormalities; XVIII—Symptoms, signs and abnormal clinical and laboratory findings, not elsewhere classified; XIX—Injury, poisoning and certain other consequences of external causes; XX—External causes of morbidity and mortality; XXI—Factors influencing health status and contact with health services. ** Referred to by the doctor-expert in the record (based on complaints) of the consultation and extracted from the text.

**Table 3:** Main causes of 5776 sick leaves (ICD diagnosis code or range).

P. *	ICD Diagnosis Code or Range	n	%	C % **
1	Major depressive disorder, recurrent	1131	19.6	19.6
2	Major depressive disorder, single episode, moderate	635	11.0	30.6
3	Dorsalgia	547	9.5	40.0
4	Convalescence after surgery	411	7.1	47.2
5	Shoulder lesions	254	4.4	51.6
6	Disorder of conjunctiva	159	2.8	54.3
7	Malignant neoplasm	158	2.7	57.0
8	Alcohol dependence	132	2.3	59.3
9	Arthropathies	103	1.8	61.1
10	Injuries of the knee and leg	96	1.7	62.8
11	Personality and behavioural disorders	81	1.4	64.2
12	Diseases of the veins and vessels and lymph nodes	80	1.4	65.6
13	Injuries of the ankle and foot	79	1.4	66.9
14	Other joint disorders	72	1.2	68.2
15	Disorder of nerves, roots and nerve plexuses	61	1.1	69.2
16	Ischemic diseases of the heart	58	1.0	70.2
17	Superficial trauma of the wrist and hand	57	1.0	71.2
18	Acute infections of the upper respiratory tract	54	0.9	72.2
19	Benign neoplasm	53	0.9	73.1
20	Diabetes Mellitus	51	0.9	74.0
21	Calculus of kidney	49	0.8	74.8
22	Disorders of the gallbladder, biliary tract and pancreas	49	0.8	75.7
23	Influenza (flu) and pneumonia	48	0.8	76.5
24	Hypertensive diseases	45	0.8	77.3
25	Diseases of the inner ear	36	0.6	77.9
26	Other local skin and subcutaneous tissue infections	36	0.6	78.5
27	Chronic diseases of the lower respiratory tract	32	0.6	79.1
28	Episodic and paroxysmal disorders	32	0.6	79.6
29	Pain and other conditions related to the female genital organs and the menstrual cycle	32	0.6	80.2
30	Dermatitis and eczema	30	0.5	80.7
31	Person in contact with health services due to other circumstances	29	0.5	81.2
32	Inflammatory polyarthropathy	29	0.5	81.7
33	Diseases of the esophagus, stomach and duodenum	27	0.5	82.2
34	Symptoms and signs that involve the digestive system and abdomen	27	0.5	82.6
35	Diseases of the oral cavity of the salivary glands and jaws	26	0.5	83.1
36	Other diseases of the upper respiratory tract	26	0.5	83.5
37	Other diseases of the urinary system	26	0.5	84.0
38	Other diseases of the intestines	26	0.5	84.4
39	Symptoms and general signs	26	0.5	84.9
40	Cerebrovascular diseases	24	0.4	85.3
41	Other viral diseases	24	0.4	85.7
42	Other forms of heart disease	24	0.4	86.1
43	Hernia	23	0.4	86.5
44	Organic mental disorders, including symptomatic disorders	22	0.4	86.9
45	Injuries of the forearm and elbow	22	0.4	87.3
46	Injuries of the shoulder and arm	22	0.4	87.7
47	Schizophrenia, schizotypal disorders and delusional disorders	21	0.4	88.0
48	Other bacterial diseases	20	0.3	88.4
49	Viral fevers transmitted by arthropods and hemorrhagic viral fevers	19	0.3	88.7
50	Obesity and other types of hyperalimentation	18	0.3	89.0
51	Other external causes of accidental injuries and falls	17	0.3	89.3
	Other (99 categories)	617	10.7	100
	Total	5776	100	

* Position; ** Cumulative percentage.
